# Scissors and curette supply solution for cutaneous sarcoid papules and nodules

**DOI:** 10.1016/j.jdcr.2024.10.029

**Published:** 2024-11-24

**Authors:** Bita Tristani-Firouzi, Elliott D. Herron, Christopher M. Hull, Mark D. Herron

**Affiliations:** a University of Utah School of Medicine, Salt Lake City, Utah; b Heersink School of Medicine, University of Alabama at Birmingham, Birmingham, Alabama; c Department of Dermatology, University of Utah, Salt Lake City, Utah; d Herron Dermatology and Laser, Montgomery, Alabama

**Keywords:** sarcoid, surgery

## Clinical challenge

**Fig 1:**
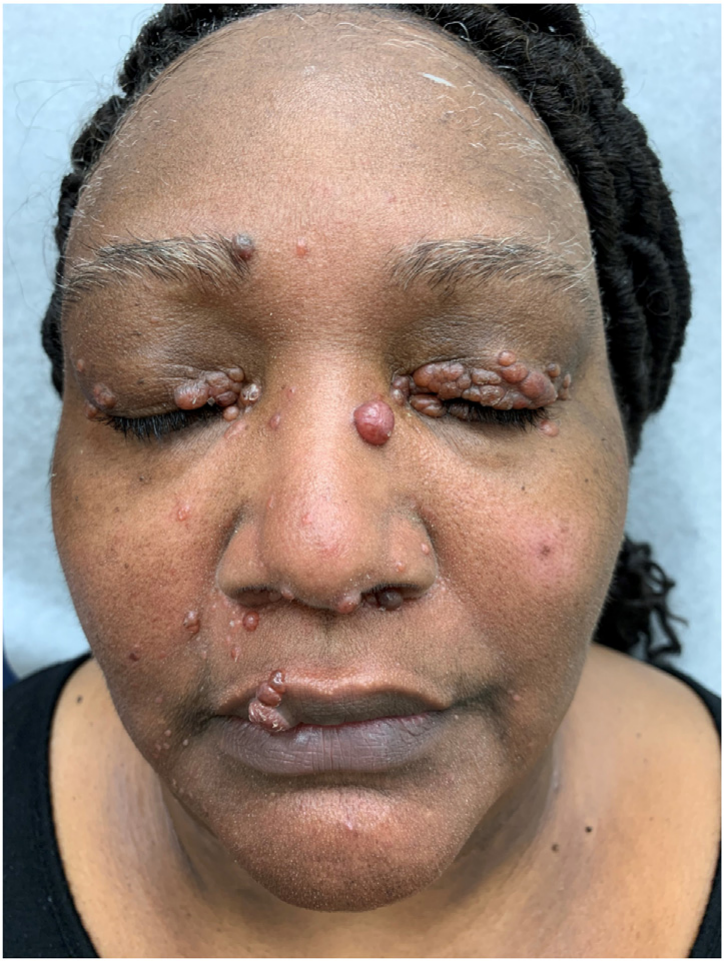
Multiple papules covering face and eyelids.

Sarcoidosis is an idiopathic granulomatous disease that affects many organ systems including the skin (Fig 1). These lesions may present as papules, plaques, subcutaneous nodules, lupus pernio, erythema nodosum, ulcers, and alopecia.^1^ Nodular cutaneous sarcoidosis is often refractory to medical treatments. It is our observation that facial nodules negatively affect patients. Topical treatments may fail to clear nodules and have side effects such as hypopigmentation. Intralesional and systemic treatment for facial sarcoidosis may not clear nodules and can have adverse effects.^2^^,^^3^

## Solution

We have employed a simple technique to remove sarcoid nodules on the face with minimal scarring. Sarcoid nodules are soft and friable making removal with scissors, curettage, and hyfrecation straightforward. We share our experience with the case of a 56-year-old African American woman having facial sarcoidosis for 5 years refractory to topical and systemic medications.

Our technique begins as first we prepare the sites with local anesthesia. We perform snip excision at the roof of the papule or nodule with Eyelid or Iris scissors. Unroofing the nodule with scissors reveals a base of granulomatous tissue. Next, we remove the base of the granuloma with gentle curetting or removing granulomatous tissue with a cotton tipped applicator. Finally, hyfrecation using PSS High Frequency Desiccator (settings 3-4) establishes hemostasis. Smaller papules on the cheek are curetted gently as one would curette molluscum papules. Particular care is taken in treating periorbital papules. Those papules are snipped with Eyelid scissors and curetted with a 1-mm diameter curette. The lesions are then wiped with 2 × 2-cm gauze to remove the granulomatous tissue. Pressure and hyfrecation (setting 2-3) is then applied for hemostasis. Sites were allowed to heal via second intention with immediate postprocedure care of petroleum jelly application.

Pigmentary changes, scarring, and keloid formation are possible with any cosmetic procedure within patients of all skin types. We chose snip excision of selected facial regions over 5 visits spanning a 6-months’ time. Choosing treatment of several papules and nodules across multiple visits afforded the opportunity to assess scarring and pigmentary changes. We tested nodules on the nose and lips, then central face, and lastly periorbital face.

**Fig 2:**
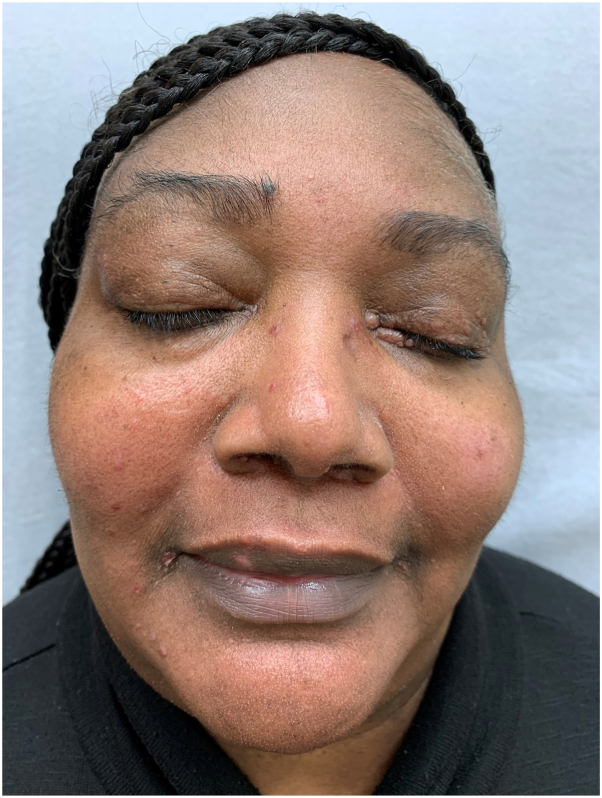
Four months follow-up.

**Fig 3:**
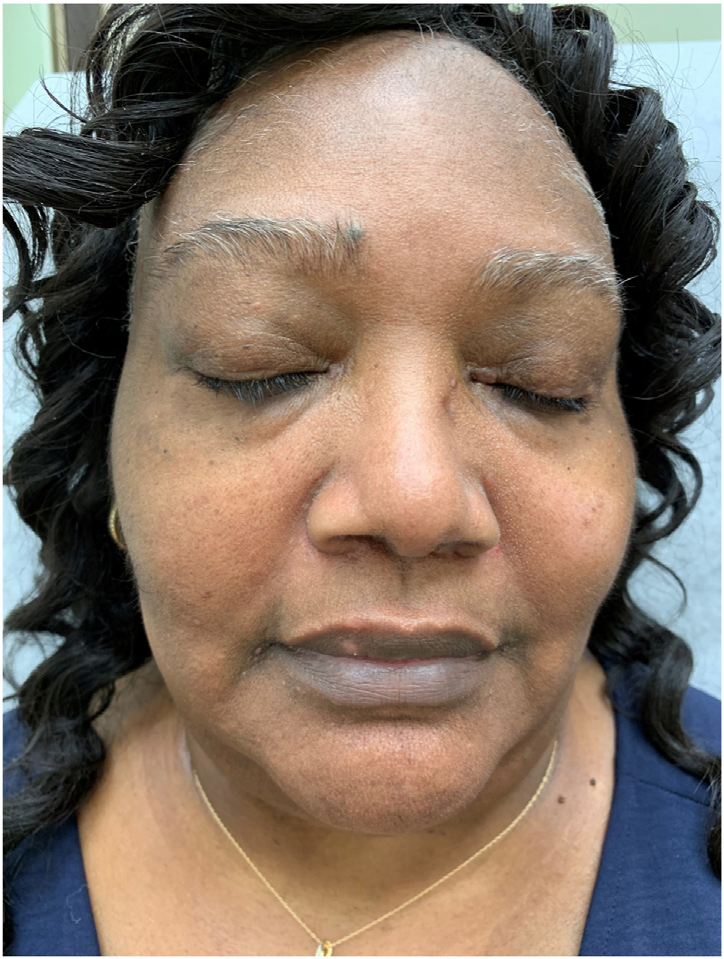
Twelve months follow-up.

Our results show the dissolution of nodules. The cosmetic appearance is acceptable with minimal hyperpigmentation and scarring across a 4 month and 12-month timeframe (Figs 2, 3). Although scissor snipping does not treat the systemic nature of sarcoidosis, it provides an elevation of self-esteem in these patients and ultimately leads to improved quality of life. Using a standard technique of scissor snipping provides safe and satisfactory results for patients with facial sarcoidosis.

## Conflicts of interest

None disclosed.
